# Stabilization of breast cancer xenograft tumour neovasculature by angiopoietin-1

**DOI:** 10.1038/sj.bjc.6600082

**Published:** 2002-02-12

**Authors:** S Tian, A J Hayes, L J Metheny-Barlow, L-Y Li

**Affiliations:** Department of Oncology, Georgetown University Medical Center, 3970 Reservoir Road, NW, Washington DC 20007, USA

**Keywords:** angiopoietin, endothelial cells, smooth muscle, pericyte, tumour, vasculature

## Abstract

Angiopoietin-1 is a promoter of physiological vasculogenesis and angiogenesis because it induces vascular branching and smooth muscle recruitment to newly formed blood vessels. However, angiopoietin-1 expression in tumours appears to be uncommon, and angiopoietin-1 overexpression in cancer cells has been reported to lead to inhibition of xenograft tumour growth. We report here that angiopoietin-1 overexpression resulted in stabilization of tumour edge-associated blood vessels, as it prevented vessel dilation and dissociation of smooth muscle cells from existing vessels. In addition, angiopoietin-1 stimulated an infiltration of mesenchymal cells into the tumours, such that the coverage of microvessels by pericytes increased markedly, and the cancer cells were separated into small masses by the host stroma. The rates of both cancer cell proliferation and apoptosis decreased significantly in the presence of angiopoietin-1. Tie2, the receptor for angiopoietin-1, was found to be present in vascular smooth muscle cells in culture in addition to endothelial cells. These findings suggest that a vascular stabilization effect of angiopoietin-1 accounts for the inhibition of tumour growth.

*British Journal of Cancer* (2002) **86**, 645–651. DOI: 10.1038/sj/bjc/6600082 www.bjcancer.com

© 2002 Cancer Research UK

## 

Interactions between endothelial cells and supporting mesenchymal cells (smooth muscle cells and pericytes) are essential to blood vessel development and function. Endothelial cells are in contact with these supporting cells via functional gap junctions, separated only by a fenestrated basement membrane (Bruns and Palade, 1968; Spitznas and Reale, 1975; Spagnoli *et al*, 1981; Leiser *et al*, 1985). The association of these cells is critical for vascular stabilization, and is accompanied by the inhibition of endothelial cell proliferation (Folkman and D'Amore, 1996; Hanahan and Folkman, 1996). Disruption of this endothelial/mesenchymal cell association is seen in disease states such as angiogenesis in cancer (Carmeliet and Jain, 2000) and restenosis after coronary angioplasty (Orford *et al*, 2000).

Angiopoietin-1 (Ang1) and the antagonistic ligand angiopoietin-2 (Ang2) are important vascular regulatory molecules whose functions have been the subjects of intensive investigation. Ang1 gene expression *in utero* is closely associated with vascular endothelial cell growth factor (VEGF, initially known as vascular permeability factor, VPF (Dvorak *et al*, 1991)) and in physiological angiogenic processes in adults such as ovulation (Maisonpierre *et al*, 1997). Null mutation of the Ang1 gene in transgenic animals led to failure of endothelial cells to form stable associations with supporting mesenchymal cells and failure of primitive capillaries to develop into a mature branched network (Suri *et al*, 1996). Transgenic Ang1 over-expression resulted in increased vascular branching (Suri *et al*, 1998). While overexpression of VEGF also led to increased vascular branching, the vessels induced by VEGF were leaky. By contrast, the vessels induced by Ang1 were not leaky and resisted leakage caused by inflammatory agents, due to markedly enhanced association of smooth muscle cells with the endothelium. Co-expression of Ang1 and VEGF had an additive effect on angiogenesis but gave rise to leakage-resistant vessels typical of Ang1 as seen when Ang1 was overexpressed alone (Thurston *et al*, 1999, 2000).

*In vitro*, Ang1 protects against endothelial cell apoptosis, and induces vascular sprouting and capillary-like tubule formation in collagen (Koblizek *et al*, 1998; Hayes *et al*, 1999; Holash *et al*, 1999; Papapetropoulos *et al*, 1999). Ang1 has been shown to interact with a receptor tyrosine kinase, Tie2, which is largely limited to endothelial cells (Dumont *et al*, 1993; Maisonpierre *et al*, 1993; Davis *et al*, 1996; Suri *et al*, 1996). Null mutation of the Tie2 gene gave rise to essentially the same phenotype seen when Ang1 was knocked out (Sato *et al*, 1995). It has also been reported that Ang1 as well as Ang2 were direct substrates in endothelial cell adhesion mediated by integrins (Carlson *et al*, 2001). Many studies have investigated the expression patterns of Ang1, other angiopoietins, and Tie2 in a variety of tumours, including cancers of the breast (Hayes *et al*, 2000; Currie *et al*, 2001), brain (Stratmann *et al*, 1998; Zagzag *et al*, 1999; Eggert *et al*, 2000), liver (Tanaka *et al*, 1999), ovary (Martoglio *et al*, 2000), lung (Takahama *et al*, 1999; Wong *et al*, 2000), Kaposi's sarcoma and cutaneous angiosarcoma (Brown *et al*, 2000), thyroid gland (Bunone *et al*, 1999), and leukaemia (Kukk *et al*, 1997). These studies revealed a complex pattern of expression of the angiopoietins and Tie2 that is consistent with an important role for Ang2 and VEGF in the destabilization of vasculature and initiation of tumour angiogenesis. In contrast the role of Ang1 in cancer was less clear.

We recently reported (Hayes *et al*, 2000) that overexpression of Ang1 in an MCF-7 xenograft model of tumour angiogenesis resulted in a retardation of tumour growth and the extent of inhibition correlated to the extent of Ang1 overexpression. Others also reported a similar inhibition of colon cancer xenograft tumour growth (Ahmad *et al*, 2001). We have now analyzed the MCF-7 xenograft tumours to assess the effect of Ang1 overexpression on the structure of the tumour vasculature and the proliferation of the cancer cells. We report here that Ang1 overexpression prevented the dissociation of smooth muscle cell from the endothelium of the tumour edge-associated blood vessels and the dilation of these vessels. It also induced an influx of stromal cells into the tumours, which greatly enhanced the coverage of microvessels by pericytes. The rates of cancer cell proliferation and apoptosis both declined significantly. These findings are consistent with the view that stabilization and maturation of tumour neovasculature induced by Ang1 has an inhibitory effect on tumour growth.

## MATERIALS AND METHODS

### Tumour model

Female athymic nude mice were ovariectomized and oestrogen-pellet supplemented (Taconic Farms, Germantown, NY, USA). Human breast cancer cell line MCF-7 was stably transfected with full length Ang1 gene, and the secretion of Ang1 into the conditioned media was determined as described (Hayes *et al*, 2000). *In vitro* culture, harvest and inoculation of 5×10^6^ cells in the region of the mammary fat pads of the mice was performed as described (Hayes *et al*, 2000). Tumour volumes were measured in a blinded manner. Tumours were dissected post mortem, bisected and half was immediately frozen in liquid nitrogen and stored at −80°C. The remaining tumour samples were fixed with 2% formaldehyde/0.2% glutaraldehyde for 4 h, washed in PBS, and embedded in paraffin. Ang1 expression in the xenograft tumours was determined by immunostaining paraffin sections (0.5 μm) with an antibody to the N-terminus of Ang1 (L41309M, kindly provided by Regeneron Inc., Tarrytown, NY, USA). A high standard of animal ethics was applied in carrying out this investigation. We assure that the animal ethics meet the standards required by the current UKCCCR Guidelines (Workman *et al*, 1998).

### Determination of microvessel density, peripheral vessel density, and vessel lumen size

Three tumour specimens of medium size from each experimental group were analyzed. Three random sections from each specimen were prepared. The sections were immunostained for endothelial cells with a rat monoclonal antibody to CD31 (M13.2, PharMingen, San Diego, CA, USA). Microvascular density was determined by manually counting CD31-positive vessels in 5∼15 most vascularized areas of each section under 400× magnification as described (Weidner and Folkman, 1996). Peripheral vascular density was measured by counting CD31-positive vessels in the stroma at the edge of the tumour. All edge-associated vessels were counted in each section under 400× magnification. The sizes of the lumen of the tumour peripheral vessels were determined by measuring the length and width of the cross sections of CD31-positive vessels, using the equation for elliptical areas S=π*(1/2 length)*(1/2 width).

### Determination of cancer cell proliferation and apoptosis rates

Cells undergoing proliferation at the time of sample collection were identified by immunostaining the tumour sections for proliferating cell nuclear antigen (PCNA) using a monoclonal antibody (PC-10, Santa Cruz Biochemicals, Santa Cruz, CA, USA) (Hall *et al*, 1990). The number of PCNA-positive cancer cells and the total number of cancer cells in at least nine randomly selected areas of each section were manually counted under 400× magnification. Three tumours per experimental group and three random sections per tumour were analyzed. Cell apoptosis rate was determined from tumour sections subjected to *in situ* TUNEL assay (Gavrieli *et al*, 1992) by using a cell death detection kit (Roche Biochemicals, Mannheim, Germany). The number of apoptotic cells was determined in a manner similar to the determination of microvessel density. Thus, the highest number of BrdU-positive cells in at least nine most apoptotic areas in each section were manually counted. Three tumours per experimental group and three random sections per tumour were analyzed.

### Determination of vascular smooth muscle-endothelial cell ratio

Tumour sections were immunostained for smooth muscle cells, using a monoclonal antibody to mouse α-smooth muscle actin (1A4, Sigma, St. Louis, MO, USA) and an FITC-conjugated anti-mouse IgG (Sigma), and for endothelial cells, using a rat monoclonal antibody to mouse CD31 and a TRITC-conjugated anti-rat IgG (Sigma). A mouse-on-mouse blocking kit (Vector, Burlinghame, CA, USA) was used to block non-specific interactions when anti-mouse antibodies were used. The cells were visualized under an Olympus confocal laser scanning microscope. Computer-assisted image analysis of the photographs of the blood vessels was carried out by using an image analysis software Optimas 5.2 (Media Cybernetics, Baltimore, MD, USA), which allows the recognition of different colours and the measurement of the areas occupied by each colour.

### Analysis of mesenchymal cell infiltration

Paraffin-embedded tumour sections were immunostained for endothelial cells with biotinylated *Griffonia* (Bandeiraca) *simplicifolia* lectin I isolectin B4 (Vector), and for smooth muscle cells with the anti-α-smooth muscle actin.

### Western analysis of Tie2 expression

Cell lysates were prepared from semi-confluent cultures of human umbilical cord vein endothelial cells (HUVEC; Clonetics, NC, USA), human coronary artery smooth muscle cells (CASMC; Clonetics), human dermal fibroblast cells (Clonetics), adult bovine aortic endothelial cells (ABAE; a gift from Peter Bohlen of ImClone, NY, USA), Chinese hamster ovary (CHO) cells (ATCC, American Type Culture Collection, Manassas, VA, USA), human breast cancer cell line MDA-MB-231 cells (ATCC), and the Ang1 transfected MCF-7 cells (clone 128), as well as vector transfected control cells, as indicated. The cell lysates were subject to SDS–PAGE and Western blotting analysis by using an anti-Tie2 antibody (sc-324, Santa Cruz Biochemicals).

## RESULTS

### Inhibition of breast cancer MCF-7 xenograft tumour growth by Ang1 overexpression

**Figure 1:**
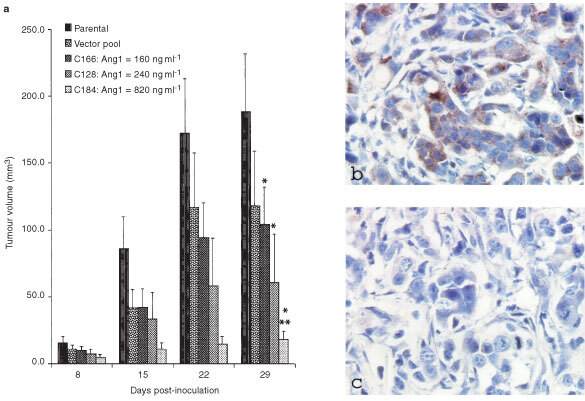
Inhibition of MCF-7 tumour growth by Ang1 overexpression. (**a**) The bars represent the average volumes (±s.e., *n*=10) of xenograft tumours formed by MCF-7 cell lines, in the order that they appear in each group: parental MCF-7, vector-transfected, Ang1-overexpressing clone-166, clone-128, and clone-184. *P<0.05 (ANOVA) compared with the parental MCF-7 control. **P<0.05 compared with the vector-transfection control. (**b**,**c**) Immunostaining of retrieved xenograft tumours for Ang1 production by Ang1-transfected cancer cells (**b**) and the control MCF-7 cells (**c**).

We reported previously that tumours formed by Ang1-transfected MCF-7 cells in nude mice grew at a much slower rate than did the parental or vector-transfected cells (Hayes *et al*, 2000). Three Ang1-overexpressing clones are shown, together with the parental MCF-7 and the vector-transfection controls. Ang1 is produced by these cell lines as a secreted protein. The yields of Ang1 in the conditioned media of clone-166, clone-128, and clone-184 are, respectively, 160 ng ml^−1^, 240 ng ml^−1^, and 820 ng ml^−1^. The higher level of Ang1 production by the cells, the slower the growth rate of the tumours formed by the cells ( Figure 1a

). The tumours were retrieved at the end of the experiment, and Ang1 expression levels in the tumours assessed by immunostaining. A significant proportion of the Ang1-transfected cancer cells were producing Ang1 in the tumours (Figure 1b). In comparison, little Ang1 was detectable in the control MCF-7 tumour (Figure 1c).

### Ang1 stabilizes the tumour edge associated blood vessels and prevents the dissociation of smooth muscle cells from endothelial cells

Intratumoural microvessel density was similar between Ang1 over-expressing tumours and controls ( Table 1

**Table 1:**
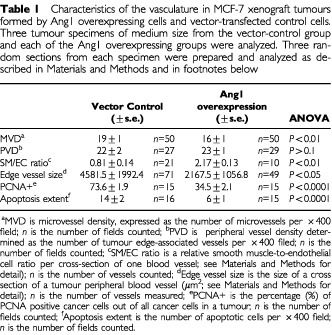
Characteristics of the vasculature in MCF-7 xenograft tumours formed by Ang1 overexpressing cells and vector-transfected control cells. Three tumour specimens of medium size from the vector-control group and each of the Ang1 overexpressing groups were analyzed. Three random sections from each specimen were prepared and analyzed as described in Materials and Methods and in footnotes below

), despite the much decreased tumour growth rate seen with Ang1 overexpression. In addition, there was no discernible change in the density of the tumour edge-associated blood vessels (Table 1). However the morphology of the tumour edge-associated vessels was altered strikingly by Ang1 over-expression. Tumours formed by the parental MCF-7 cells typically possessed extremely ectatic blood vessels with large calibre lumens at the edge of the tumour. Such dilated vessels were absent in tumours overexpressing Ang1. The edge-associated vessels in the stroma of MCF-7 tumours were poorly supported by smooth muscle cells. This was evident from double immunostaining of the endothelial cells (CD31 and TRITC) and smooth muscle cells (αSMA and green FITC) of these vessels. Many edge-associated vessels in the parental MCF-7 tumours had a thin and discontinuous layer of smooth muscle ( Figure 2a

**Figure 2:**
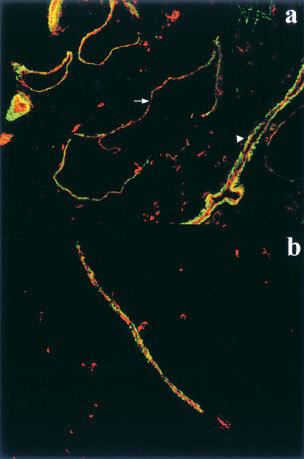
Ang1 prevents dissociation of smooth muscle cells from tumour edge-associated vessels. Confocal microscopic images of tumour edge-associated vessels were obtained by double immuno-fluorescent staining. Original magnification x200. Endothelial cells (red) were immunostained for CD31 and visualized with TRITC. Smooth muscle cells (green) were immunostained for α-actin and visualized with Green-FITC. (**a**) Many peripheral blood vessels in the control MCF-7 tumours are highly dilated and poorly supported by smooth muscle (arrow). Some blood vessels appear to have a normal smooth muscle layer (arrow head). (**b**) A typical edge-associated vessel in Ang1-overexpressing tumours showing an undilated lumen with good smooth muscle support.

). Some of the edge-associated vessels retained a normal layer of smooth muscle. In contrast, most of the edge-associated vessels in Ang1-overexpressing tumours were well supported by a continuous layer of smooth muscle cells (Figure 2b). We quantitatively determined smooth muscle-to-endothelial cell ratio in the walls of the tumour edge-associated vessels by measuring the areas occupied by the red colour, which represents endothelial cells, and the areas occupied by the green colour, which represents smooth muscle cells. This smooth muscle-to-endothelial cell ratio was nearly three times higher in the peripheral blood vessels in the Ang1-overexpressing tumours, as compared with that in the control tumours (Table 1). We then compared the lumen sizes of the peripheral vessels in the two groups of tumours positively identified by CD31 staining. We assumed a typical elliptic shape of the cross-section of the vessel lumen. The average lumen size of the peripheral blood vessels in the control tumours is more than twice that of the Ang1-overexpressing tumours (Table 1) suggesting a correlation between dissociation of smooth muscle cells and dilation of the tumour blood vessels. These results indicate that Ang1 stabilized the existing blood vessels at the tumour edge by preventing the dissociation of smooth muscle cells from the endothelium.

### Ang1 enhances microvessel coverage by pericytes

**Figure 3:**
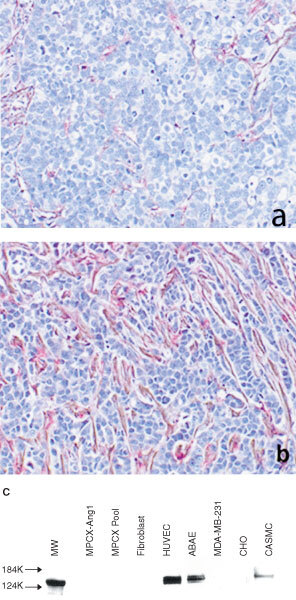
Ang1 overexpression causes marked infiltration of smooth muscle cells into tumours. (**a**) Microscopic image of a section of control MCF-7 tumours. (**b**) Microscopic image of a section of Ang1-overexpressing tumours. Tumour sections were immunostained for smooth muscle cells with antibody to α-smooth muscle actin and DAB substrate (brown) and endothelial cells with biotinylated *Griffonia* (*Bandeiraca*) *simplicifolia* lectin I isolectin B4 and Vector Red (red), and counter stained with Gill's haematoxylin (blue). Original magnification ×100. (**c**) Tie2 expression in vascular smooth muscle cells and endothelial cells in culture. Total cell lysates prepared from confluent cell cultures were analyzed by Western blotting. MW, molecular weight standard; MPCX-Ang1, Ang1-transfected MCF-7 cells; MPCXPool, vector-transfected MCF-7 cells. Fibroblast, human dermal fibroblast cells; HUVEC, human umbilical cord vein endothelial cells; ABAE, adult bovine aortic endothelial cells; MDA-MB-231, human breast cancer cell line; CHO, Chinese hamster ovary cells; CASMC; human coronary artery smooth muscle cells.

We examined the blood vessels inside the tumours and found that there was a marked infiltration of SMA-positive cells into the Ang1-overexpressing MCF-7 tumours. The tumours formed by parental MCF-7 cells consisted of a large, continuous mass of cancer cells with microvessels. The vessels were scarce, thin, and occasionally covered by SMA-positive pericytes ( Figure 3a

). The interior of the tumours formed by the Ang1-transfected MCF-7 cells, however, was remarkably different. The cancer cells in these tumours formed small, isolated masses separated by the host stroma made of an abundant number of SMA-positive cells that were almost always associated with microvessels (Figure 3b). The results indicate that the number of pericyte-associated microvessels increased significantly in the presence of Ang1. Ang1 thus facilitated the maturation of the tumour vasculature by inducing an influx of mesenchymal cells into the tumour and promoted the formation of blood vessels well-supported by smooth muscle cells and pericytes. Tie2, the receptor for Ang1, has been shown to be predominantly an endothelial cell-specific gene. The marked infiltration of smooth muscle cells into the Ang1-overexpressing MCF-7 tumours, however, prompted us to determine whether vascular smooth muscle cells also expressed Tie2. Western blotting analysis of a number of different types of cells indicated that cultured human vascular smooth muscle cells express Tie2 under the experimental conditions, although the level of expression is much lower than that in endothelial cells (Figure 3c). This suggests that Ang1 may be able to act directly on smooth muscle cells to facilitate their infiltration into the tumours.

### Decreased cancer cell proliferation and apoptosis in Ang1-overexpressing tumour

**Figure 4:**
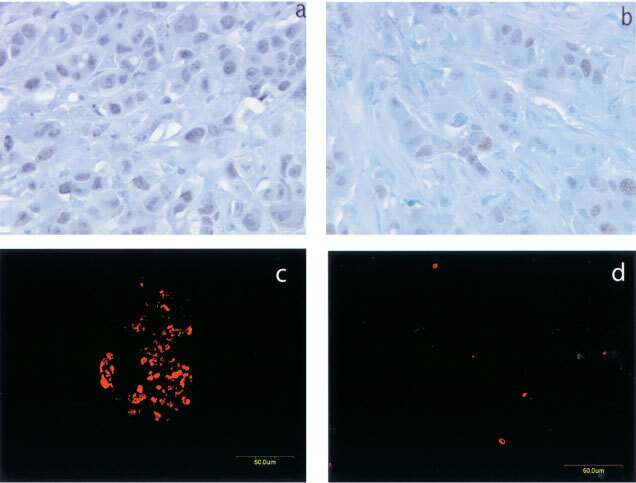
Ang1-overexpression coincides with a decrease of cancer cell proliferation and apoptosis. Tumour sections were immunostained for PCNA with DAB-Ni substrate (black), counter stained with Gill's haematoxylin (blue). Original magnification ×400. (**a**) Microscopic image of a section of MCF-7 tumours. (**b**) Microscopic image of a section of Ang1-overexpressing tumours. Apoptotic cells are identified by *in-situ* TUNEL labelling visualized with TMR-Red (red). Original magnification ×200. (**c**) Confocal microscopic image of a typical apoptotic cell population in the control parental MCF-7 tumours. (**d**) Confocal microscopic image of the Ang1-overexpressing tumours.

We investigated the growth rate of the Ang1-transfected cancer cells in the tumours as well as in cell cultures. The tumour sections were stained for proliferating cell nuclear antigen (PCNA). The PCNA-positive cells were counted. Most of the cancer cells in the control MCF-7 tumours were undergoing proliferation ( Figure 4a

, Table 1). In contrast, a much smaller fraction of the cancer cells were growing in Ang1-overexpressing tumours (Figure 4b, Table 1). The inhibitory effect on the growth of the xenograft tumours was not a direct activity of Ang1 on cancer cells, since Ang1-transfection had no effect on the growth rate of the cancer cells in culture as assessed by an *in vitro* mitogenesis assay utilizing a colorimetric vital dye (data not shown). We then determined the impact of Ang1 on the apoptosis rate of the cancer cells by measuring BrdU incorporation into fragmented nuclear DNA molecules occurring during programmed cell death. Many cancer cells in the control MCF-7 tumours underwent apoptosis, usually in groups (Figure 4c). There were, however, no such pockets of apoptotic cells in the Ang1-overexpressing tumours (Figure 4d). We determined the average number of apoptotic cells in a given tumour section, and found that it decreased in the presence of Ang1 to about one-third that of the control group (Table 1). These results, together with the decreased PCNA staining of the cancer cells, indicate that the cancer cells in Ang1-overexpressing tumours had become relatively quiescent.

## DISCUSSION

Destabilization of existing blood vessels is an essential step in the initiation of tumour angiogenesis (Gimbrone *et al*, 1972). This study indicates that Ang1 may mediate vascular stabilization by acting on the mesenchymal component of the tumour. The action appears to result in a strengthening of the existing blood vessels surrounding a tumour, and an enhancement of smooth muscle cell and pericyte population in the tumour. These effects apparently give rise to a negative impact on tumour growth, as we and others have observed (Hayes *et al*, 2000; Ahmad *et al*, 2001).

The process of tumour angiogenesis has been likened to a wound that fails to heal with a continuous cycle of vascular sprouting, exudation of tissue fluid, and invasion of mesenchymal cells (Dvorak, 1986). As a result, the tumour vessels have irregular diameters, frequent intussusception and irregular branching patterns. They have incomplete basement membranes and discontinuous pericyte coatings (Carmeliet and Jain, 2000). The aberrant structure and function of the tumour vascular system not only allows but stimulates the ongoing angiogenic processes of capillary sprouting and exudation of stroma as the tumour continues to grow.

The histopathological analysis of the MCF-7 xenograft tumours revealed a number of important activities of Ang1. First, Ang1-overexpression is correlated to stabilization of the vessels at the leading edge of tumour growth. This is evident from the prevention of vessel dilation and the maintenance of regular pericyte support of the vessel wall. Second, Ang1-overexpression coincides with the infiltration of an excess of mesenchymal cells into the tumour, comprising principally, though not exclusively, smooth muscle cells. These cells become associated with endothelial cells to form a vascular system surrounding nests of tumour cells. Third, the rates of both cancer cell proliferation and apoptosis are substantially diminished in the Ang1 overexpressing tumours. This cannot be ascribed to a direct inhibitory activity of Ang1 on the cancer cells, as MCF-7 cell growth rate in culture is not altered by overexpression of Ang1.

The important question is why the effects of Ang1 overexpression in tumours result in a retardation of growth. It is plausible that the stabilization effect of Ang1 on the ectatic vessels at the periphery of the tumour is of particular significance. Recent studies have shown that an angiogenic response induced by overexpression of VEGF/VPF involved the formation of highly permeable, enlarged, thin-walled, and pericyte-poor variations of blood vessels. These vessels developed from pre-existing microvessels after pericyte detachment and basement membrane degradation (Pettersson *et al*, 2000). They are similar to those seen here in the periphery of the parental MCF-7 tumours, which are known to express VEGF (Heer *et al*, 1998; Ruohola *et al*, 1999). It is known that Ang1 can act to prevent the permeability effects of VEGF by its effect on endothelial cell-pericyte association (Thurston *et al*, 1999, 2000). It is therefore reasonable to surmise that the crucial event in the ongoing growth of a tumour is the destabilization of native vessels at the tumour periphery as a result of VEGF expression, producing ectatic and highly permeable vessels. Ang1 appears to be able to prevent this process presumably by stabilizing the pericyte-endothelial cell association. This in turn results in a negative effect on the growth of the tumour cells which accounts for the changes in rates of tumour cell proliferation and apoptosis. It has long been recognized that the growth rate of a tumour is not proportional to the growth rate of the cancer cells but to that of the blood vessels (Tannock, 1968). It is plausible that tumour growth diminishes when the tumour is unable to initiate angiogenesis from a vasculature stabilized by Ang1.

The molecular mechanism by which Ang1 exerts its effects on the endothelial-mesenchymal association is not clear. The current hypothesis is that, since Tie2, the receptor for Ang1, is primarily limited to endothelial cells, the recruitment of mesenchymal cells to a newly formed blood vessel is the result of a paracrine loop between endothelial cells, which respond to Ang1, and the mesenchymal cells which respond to intermediary signalling molecules released by the endothelial cells (Folkman and D'Amore, 1996). Putative intermediary molecules have been suggested but such a paracrine loop has yet to be identified. Our findings that smooth muscle cells express Tie2 in culture, and that a large number of smooth muscle cells infiltrate into Ang1-overexpressing tumours suggest a direct role of Ang1 on mesenchymal cells under certain conditions to facilitate vascular stabilization. It is important to point out that our observations are limited to an experimental breast cancer model, although they have been reflected in a study on a colon cancer model by others (Ahmad *et al*, 2001). Whether Ang1 plays a similar role under clinical conditions remains unclear. Further studies should be directed to explore this interesting role. The therapeutic potential of Ang1 either as recombinant proteins or delivered by means of gene transfer should also be investigated.

Current anti-angiogenic approaches focus on targeting endothelial cells. Our study suggests that mesenchymal vascular components should also be emphasized. Tumour angiogenesis is not simply reliant upon the division of endothelial cells but also involves processes such as vascular destabilization and vascular permeability changes. The identification of Ang1 as a molecule that may alter these processes and so retard tumour growth opens new avenues for intervention on the tumour angiogenic process.
